# Genetic Subtraction Profiling Identifies Candidate miRNAs Involved in Rice Female Gametophyte Abortion

**DOI:** 10.1534/g3.117.040808

**Published:** 2017-05-19

**Authors:** Liyu Yang, Ya Wu, Wenliang Wang, Bigang Mao, Bingran Zhao, Jianbo Wang

**Affiliations:** * State Key Laboratory of Hybrid Rice, Department of Plant Science College of Life Sciences, Wuhan University, 430072 China; † Hunan Hybrid Rice Research Center, Changsha 410125, China

**Keywords:** ovule development, female gametophyte abortion, high-throughput sequencing, miRNA, rice

## Abstract

The female gametophyte is an important participant in the sexual reproduction of plants. The molecular mechanism of its development has received much attention in recent years. As important regulators of gene expression, miRNAs have been certified to play a significant role in many biological processes of plants, including sexual reproduction. In this study, to investigate the potential regulatory effects of miRNAs on rice female gametophyte abortion, we used the high-throughput sequencing method to compare the miRNA transcriptome in ovules of a high frequency female-sterile line (*fsv1*) and a rice wild-type line (Gui 99) during ovule development. As a result, 522 known miRNAs and 295 novel miRNAs were expressed in the developing ovule of rice, while 100 known miRNAs were significantly differentially expressed between these two rice lines during ovule development. Combining with gene expression information, a total of 627 coherent target genes of these differential expressed known miRNAs between *fsv1* and Gui 99 were identified. The functional analyses of these coherent target genes revealed that the coherent target genes of differential expressed known miRNAs between the two rice lines are involved in many biological pathways, such as protein degradation, auxin signal transduction, and transcription factor regulation. These results provide us with important clues to investigate the regulatory roles of miRNAs in rice female gametophyte abortion.

Plant microRNAs (miRNAs) are short (20–24 nt), noncoding RNAs that play an essential role in regulating many biological processes, such as plant organ development, phase change from vegetative growth to reproductive growth, and response to stress (Aukerman and Sakai 2003; Achard *et al.* 2004; Jones-Rhoades and Bartel 2004; Mallory *et al.* 2004). They usually act as important negative regulators to alter the expression of genes by directing the target mRNA cleavage or translational inhibition. In plants, the miRNA gene is transcribed into pri-miRNA by RNA polymerase II at first, and then pri-miRNA is cleaved into pre-miRNA with a secondary hairpin structure by DCL1 and HYL1. Pre-miRNA is further processed into the miRNA:miRNA* complex (miRNA* refers to one strand of miRNA:miRNAcomplex which is not selected for entry into a silencing complex). Generally, only miRNAs regulate gene expression by binding to complementary target mRNAs, and the miRNA* sequence is therefore degraded (Borges and Martienssen 2015).

In recent years, there is growing evidence that the small RNA–dependent silencing pathway is involved in the sexual reproduction of plants (Olmedo-Monfil *et al.* 2010; Fei *et al.* 2016). The abnormal expression of miRNAs could lead to gametophyte developmental defects and even sterility. For example, in *Arabidopsis*, *ARF6* and *ARF8* transcripts were targeted by miR167. The expression variation of miR167 could result in the abnormal expression of *ARF6* and *ARF8*, and ultimately affect male and female reproduction (Wu *et al.* 2006).

Rice is one of the most important food crops in the world. In the current miRBase (release 21), 713 mature miRNAs were identified in rice (http://www.mirbase.org/) and many of them have been proved to be involved in a lot of important biological processes, including leaf development, pattern formation, response to stress, and sexual reproduction (Nagasaki *et al.* 2007; Zhu *et al.* 2009; Xie *et al.* 2012; Fang *et al.* 2014). Recently, deep-sequencing studies have demonstrated that miRNAs were involved in the process of rice male gamete formation, especially in the fertility transition. According to the research of Wei *et al.* (2011), 202 miRNAs were found to be expressed in rice during pollen development. Numerous miRNAs exhibited significant differential expressions between the male sterility line and its maintainer line in rice (Yan *et al.* 2015). Although recent studies have investigated the effects of miRNAs on regulating male gametophyte development of rice, few studies have focused on the roles of miRNAs in regulating the fertile female gametophyte formation.

As the core of the ovule, the female gametophyte relies utterly on the protection and nutrition provided by the ovule. The ovule and female gametophyte develop at approximately the same time and are closely connected with each other. Female gametophyte fertility is vital for sexual reproduction in rice, and female gametophyte formation involves two consecutive processes: megasporogenesis and megagametogenesis (Coelho *et al.* 2007). These processes are precisely controlled by a large number of genes expressed in the surrounding sporophytic ovule tissues or the developing female gametophyte (Pagnussat *et al.* 2007; Singh *et al.* 2011). Failure in the proper function of any step could result in female sterility. Although the molecular mechanism of female sterility is not clearly understood, based on the close relationship between miRNAs and rice male gametophyte fertility, it is probable that the small RNA pathway is involved in rice female gametophyte abortion. Recently, evidence supporting this hypothesis has been obtained from a study by Nonomura *et al.* (2007). In the developing ovule of rice, *MEL1* (MEIOSIS ARRESTED AT LEPTOTENE1, *OsAGO5c*), which is a key component of the miRNA pathway, is expressed in female germ cells. Most megaspore mother cells (MMCs) in the *mel1-1* mutant fail to enter or finish meiosis, ultimately leading to female sterility (Nonomura *et al.* 2007). This implies that small RNA pathway is required for the specification of rice female gametic cells. It is necessary to reveal the regulatory effects of the small RNA pathway on rice female germline development.

In the present study, we used high-throughput sequencing methods to analyze miRNAs and their targets during ovule development in a high frequency female-sterile rice line (*fsv1*) and a rice wild-type line (Gui 99). A total of 522 known miRNAs and 295 novel miRNAs were identified in *fsv1* and Gui 99 ovules. Numerous miRNAs exhibited distinct expression patterns in *fsv1* and Gui 99 during the ovule development. A total of 100 known miRNAs exhibited significant differential expression between the ovules of these two rice lines. The differentially expressed miRNAs between *fsv1* and Gui 99 suggested that miRNAs could be associated with rice female gametophyte abortion, and our results provide important clues to reveal the molecular mechanisms underlying ovule development and rice female gametophyte sterility.

## Materials and Methods

### Plant materials

Gui 99 and *fsv1* were used as plant materials. The *fsv1* used in this study was previously described by Zhao *et al.* (1998). Gui 99 and *fsv1* were cultivated in the greenhouse of Wuhan University, Wuhan, China. According to the developmental characteristics of the ovule and embryo sac, the formation process of the ovule was divided into nine stages by Lopez-Dee *et al.* (1999). Ovules used in this study were harvested at three developmental stages: ovule containing a MMC undergoing meiosis (stage 1), ovule containing a functional megaspore undergoing mitosis (stage 2), and ovule containing a mature female gametophyte (stage 3). These three developmental stages corresponded to ov4, ov5–ov7, and ov8–ov9 in Lopez-Dee *et al.* (1999), respectively. A1, A2, and A3 represented ovules at stage 1, 2, and 3 in Gui 99, while B1, B2, and B3 represented ovules at stage 1, 2, and 3 in *fsv1*. For extracting enough total RNA for sequencing, the ovules were pooled from at least 100 ovaries at each time point.

### Small RNA library preparation and sequencing

Total RNA was prepared by using TRIzol (Invitrogen, Burlington, Canada) according to the protocol. The concentration and purity of each RNA sample were determined by the absorbance at 260 and 280 nm. The Agilent 2100 Bioanalyzer (Agilent Technologies, Santa Clara, CA) with the Agilent RNA 6000 Nano Assay Kit were used to monitor the integrity of each RNA sample. Small RNA fragments of 18–30 nt were separated by 15% polyacrylamide gel electrophoresis. Using T4 RNA ligase, the 3′ and 5′ terminuses of fragments were ligated with Solexa adaptors. Subsequently, the ligation products were reverse-transcribed to cDNA and amplified after 15 cycles of PCR. Finally, the cDNA libraries were sequenced using Illumina HiSeq2000 (Illumina, San Diego, CA) at Beijing Genomics Institute (BGI), Shenzhen, China.

### Analysis of sequencing data

The rice genome and gene information were downloaded from the Rice Genome Annotation Project (http://rice.plantbiology.msu.edu). After high-throughput sequencing, we could get the clean reads after removing the low-quality reads, reads with 5′ adaptor primer contaminants, reads without the 3′ adaptor primer, reads without the insert tag, reads with poly-A, and reads shorter than 18 nt. Then, the resulting 18–30 nt RNAs were aligned to the rice genome using SOAP2 software. Clean small RNA sequences that mapped to rRNA, scRNA, snoRNA, snRNA, tRNA, and repeat sequences were removed based on the NCBI database (http://www.ncbi.nlm.nih.gov/) and Rfam database (http://www.sanger.ac.uk/resources/databases/rfam.html). The clean small RNA sequences assigned to the exon region and intron region of protein-coding genes were also discarded. The rice known miRNAs were obtained by a Blast search against the miRNA precursors and mature miRNAs of the rice miRNAs database available in miRBase database (21.0) (http://www.mirbase.org/). To identify novel miRNA candidates, the unannotated small RNA sequences were aligned to the rice genome to obtain miRNA precursors, and then the structural features of miRNA precursors were analyzed by MIREAP software (https://sourceforge.net/projects/mireap/). The criteria used to identify novel miRNAs were proposed by Allen *et al.* (2005).

### Differential expression analysis of miRNAs

To calculate the amount of miRNA expression, the transcripts per million (TPM) algorithm was used to normalize the read count of each identified miRNA in each sample. If the expression value of miRNA was 0 after normalization, the normalized expression value of this miRNA was set to 0.01. If the normalized miRNA expression was <1 in both samples, differential expression analysis was not performed. To get the significantly differentially expressed miRNA, we calculated the fold-change and *P*-value. The fold-change in different comparisons was calculated as follows: Fold change = log_2_ (normalized expression of miRNA in A2/A1 or A3/A2 or A3/A1 or B1/B2 or B2/B3 or B1/B3 or A1/B1 or A2/B2 or A3/B3). The calculation method for *P*-values has been described by Audic and Claverie (1997). miRNA that satisfied the requirement of |fold change log_2_| > 1 and *P*-value <0.05 were defined as significantly differentially expressed miRNA.

### Prediction and functional analysis of target genes of differentially expressed known miRNAs

According to the miRNA target predicting proposed by Allen *et al.* (2005) and Schwab *et al.* (2005), the sequences of known miRNAs were aligned to the rice genome. psRobot software (http://omicslab.genetics.ac.cn/psRobot/) and TargetFinder software (http://targetfinder.org/) were used to identify the putative targets of differentially expressed known miRNAs in all comparisons. Combined with information on gene expression, the predicted targets that exhibited opposite expression trends with their corresponding miRNAs in the same comparison were defined as coherent target genes. Information on coherent target genes belonging to the transcription factor families was obtained from the Plant Transcription Factor Database (http://planttfdb.cbi.pku.edu.cn/). The gene ontology (GO) annotations of these coherent target genes were obtained by using BLAST2GO (version 2.3.5) (http://www.blast2go.org/). Then we used the WEGO website (http://wego.genomics.org.cn/) to get the GO functional classification analysis of all coherent targets. To recognize the major biological functions of candidate target genes, we performed GO functional enrichment analysis. We first mapped all coherent target genes of differentially expressed known miRNAs to each term of the GO database and calculated the number of target genes for each term. Then, we used the hypergeometric test to find out the GO terms that were significantly enriched in the candidate target genes compared with the whole referenced gene background. The formula was as follows:

$$P=1-\sum_{i=0}^{m-1}\frac{\left(\begin{array}{l} M\\ i \end{array}\right)\left(\begin{array}{l} N-M\\ n-i \end{array}\right)}{\left(\begin{array}{l} N\\ n \end{array}\right)}.$$

In the formula, *N* is the number of genes with GO annotation in the whole reference gene background, *n* is the number of predicted candidate target genes in *N*, *M* is the number of genes that were annotated to a specific GO term in the whole reference gene background, and *m* is the number of candidate target genes that was annotated to a specific GO term. The Bonferroni correction was used to correct the *P*-value. GO terms that satisfied the requirement of corrected *P*-value ≤0.05 were defined as significantly enriched.

### miRNA detection by stem-loop RT-PCR and validation by quantitative real-time PCR

Total RNA was extracted from pooled ovules of *fsv1* and Gui 99 at each stage using TRIzol (Invitrogen). Then, the stem-loop RT-PCR was used to detect miRNA expression according to Varkonyi-Gasic *et al.* (2007). The DNase I–treated total RNA was reverse-transcribed by miRNA-specific stem-loop primers. The reverse transcription was performed at 16° for 30 min, 30° for 30 sec (60 cycles), 42° for 30 sec, 50° for 1 sec, and then 70° for 5 min. The rules in designing the stem-loop primers were based on Chen *et al.* (2005). The quantitative real-time PCR was carried out using the ABI Step One Plus Real-Time PCR System (Applied Biosystems) with the Thunderbird SYBR qPCR mix (Toyobo, Japan). The quantitative real-time PCR amplification reactions were performed under the following cycling parameters: 95° for 30 sec, 95° for 10 sec, 56° for 30 sec (40 cycles), and 72° for 15 sec. *U6* was used as internal control for the miRNAs. Three biological replicates and two technological replicates were implemented for each sample and the results were represented as the mean ± SD of these replicates. In this study, four novel miRNAs were randomly selected and then detected by gel electrophoresis after stem-loop RT-PCR. The primers used in stem-loop RT-PCR and quantitative real-time PCR are all listed in Supplemental Material, Table S1.

### Data availability

The miRNA sequences used in this study have been submitted to the NCBI Sequence Read Archive (SRA) Database. The accession numbers of the six SRA runs are SRR4046266, SRR4046278, SRR4046296, SRR4046297, SRR4046299, and SRR4046312 (http://trace.ncbi.nlm.nih.gov/Traces/sra/sra.cgi?view=run_browser).

## Results

### The small RNA profiles in Gui 99 and fsv1 ovules

**Figure 1:**
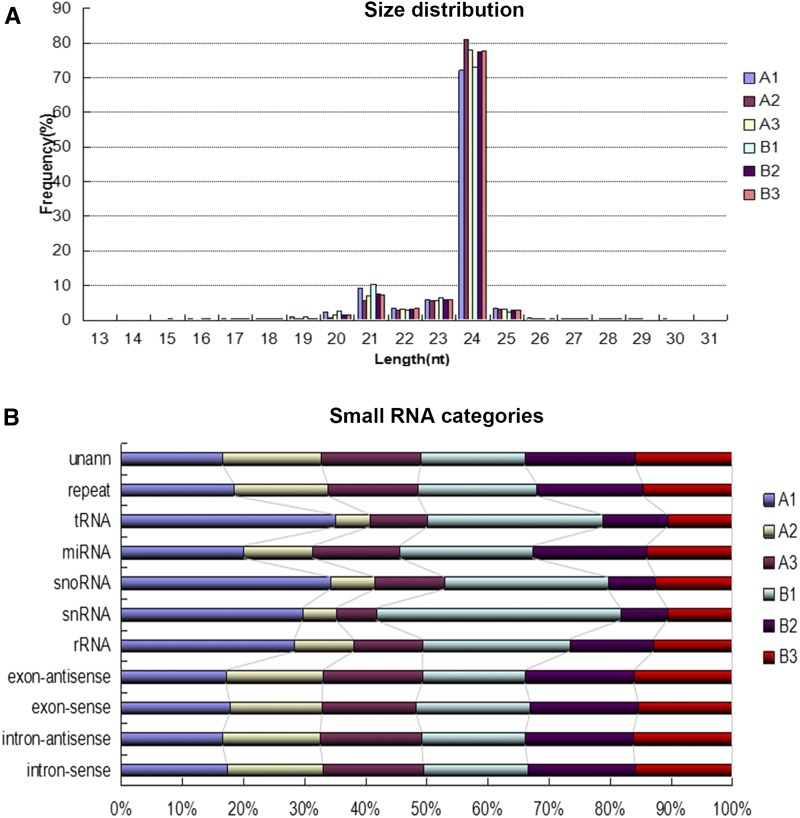
The length distribution and categories distribution of small RNAs in all six libraries. (A) The length distribution of small RNAs in all six libraries. (B) Category distribution of small RNAs in all six libraries.

In this study, ovules were harvested at three developmental stages (for developmental classification criterion, see *Materials and Methods*). A1, A2, and A3 represent ovules at stage 1, 2, and 3 in Gui 99, whereas B1, B2, and B3 represent ovules at stage 1, 2, and 3 in *fsv1*. To investigate the small RNAs correlated with rice ovule development and female gametophyte fertility, Illumina high-throughput sequencing technology was employed to identify the small RNA expression profiles of Gui 99 and *fsv1* ovules at the three developmental time points. As a result, sequencing of the A1, A2, A3, B1, B2, and B3 libraries generated 11,961,429, 10,672,237, 10,745,688, 12,339,354, 12,157,330, and 10,569,081 high-quality reads, respectively. After discarding the adaptor sequences, poly-A sequences, and sequences <18 nt, a total of 68,168,072 clean reads were generated from the six libraries (11,896,357 clean reads in A1; 10,659,126 clean reads in A2; 10,726,026 clean reads in A3; 12,213,502 clean reads in B1; 12,123,570 clean reads in B2; and 10,549,491 clean reads in B3). As shown in Figure 1A, the length distributions of small RNAs were very similar in the six libraries, and the lengths of most small RNAs ranged from 21 to 24 nt. The 24 nt small RNAs were the most abundant. The length distribution of small RNAs is consistent with previous reports (Wei *et al.* 2013; Zhang *et al.* 2016).

### Identification of known miRNAs in fsv1 and Gui 99 ovules

**Figure 2:**
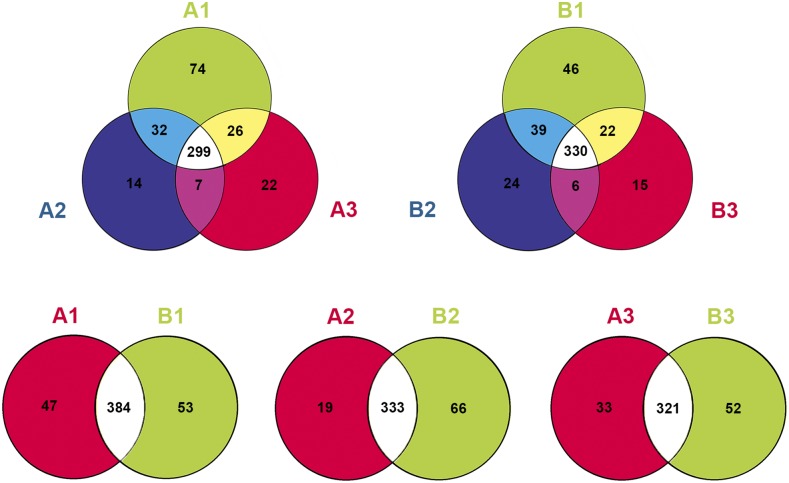
Venn diagram for known miRNAs expressed in the *fsv1* and Gui 99 ovules at three ovule developmental stages. A total of 299 known miRNAs were expressed at all three ovule developmental stages of the Gui 99 ovule, 32 known miRNAs were coexpressed in A1 and A2, seven known miRNAs were coexpressed in A2 and A3, and 26 known miRNAs were coexpressed in A1 and A3. The number of exclusively expressed known miRNA in A1, A2, and A3 was 74, 14, and 22, respectively. In *fsv1*, 330 known miRNAs were coexpressed in B1, B2, and B3. In addition, 39 known miRNAs were coexpressed in B1 and B2, six known miRNAs were coexpressed in B2 and B3, and 22 known miRNAs were coexpressed in B1 and B3. The number of exclusively expressed known miRNA in B1, B2, and B3 was 46, 24, and 15, respectively. During ovule development, the number of coexpressed known miRNA in *fsv1* and Gui 99 was 384, 333, and 321 at stage 1, stage 2, and stage 3, respectively. The number of exclusively expressed known miRNA was 47 (A1) and 53 (B1) at stage 1, 19 (A2) and 66 (B2) at stage 2, and 33 (A3) and 52 (B3) at stage 3.

By searching the Rfam and NCBI GenBank databases, the distribution of small RNAs among different categories were identified (Figure 1B and Table 1). These small RNAs included miRNA, rRNA, snRNA, snoRNA, tRNA, and so on, and ∼35.36–40.07% small RNAs were annotated. miRNAs account for almost 2.15–3.60% of the clean reads in the six libraries. The known miRNAs were obtained by a Blast search against the known miRNA precursors and mature miRNAs in miRBase database. As a result, 522 known miRNAs (431 known miRNAs in A1, 352 known miRNAs in A2, 354 known miRNAs in A3, 437 known miRNAs in B1, 399 known miRNAs in B2, and 373 known miRNAs in B3) were obtained in the six libraries (Table S2), and more than half of the known miRNAs (319 known miRNAs) exhibited different expression patterns in ovules of Gui 99 and *fsv1* during ovule development. The most abundant three families of miRNA were miR812, miR166, and miR395. Venn diagrams were used to depict known miRNAs detected in Gui 99 and *fsv1* ovules at three developmental stages (Figure 2). In Gui 99 and *fsv1* ovules, most miRNA were continuously expressed during ovule development, whereas some miRNAs were expressed at a specific stage of ovule development, suggesting that miRNAs may be involved in the regulation of rice ovule development. At the same developmental stage, more miRNAs were obtained in the *fsv1* ovule than the Gui 99 ovule. The number of coexpressed known miRNAs in *fsv1* and Gui 99 exhibited a decreasing trend during ovule development, suggesting that miRNA may be involved in regulating female gametophyte fertility mainly at the early stage of ovule development.

**Table 1:** Distribution of small RNA among different categories in six libraries

RNA Class	A1		A2		A3		B1		B2		B3	
	Unique sRNAs	Total sRNAs	Unique sRNAs	Total sRNAs	Unique sRNAs	Total sRNAs	Unique sRNAs	Total sRNAs	Unique sRNAs	Total sRNAs	Unique sRNAs	Total sRNAs
Intron-Sense	93,705	321,130	72,463	292,670	71,647	304,725	93,589	317,852	80,250	326,003	70,248	295,456
Intron-Antisense	87,076	288,612	69,657	278,111	68,927	289,678	86,616	294,167	76,383	306,892	67,277	282,079
Exon-Sense	152,382	326,027	97,274	277,595	97,205	281,847	156,502	342,075	120,143	323,921	100,450	283,083
Exon-Antisense	80,745	204,040	65,925	189,126	65,122	194,103	84,877	199,664	76,368	212,678	64,498	191,348
rRNA	34,778	217,139	17,445	73,809	18,377	86,648	26,801	183,749	20,991	104,946	18,227	98,613
snRNA	2223	9549	932	1783	1019	2105	1786	12,875	1112	2475	1209	3420
snoRNA	2835	7446	1022	1577	1243	2486	2163	5816	1049	1703	1391	2726
miRNA	3476	406,533	2250	228,656	2500	288,572	3364	439,810	2717	378,016	2395	284,682
tRNA	14,864	168,264	8851	27,314	8669	45,281	12,613	138,239	9274	50,686	8180	51,173
Repeat	1,198,302	2,883,582	918,941	24,06,842	881,419	2,296,972	1,215,740	3,045,761	1,023,658	2,710,335	877,058	2,290,521
Unannotated	2,947,881	7,064,035	2,799,703	6,881,643	2,781,868	6,933,609	2,881,541	7,233,494	3,139,472	7,705,915	2,710,295	6,766,390

### Identification of novel miRNAs in fsv1 and Gui 99 ovules

Of all the generated small RNA sequences, some small RNA sequences could be mapped to the rice genome but could not be mapped to the known miRNAs in miRBase database or other types of small RNAs. These small RNA sequences were used to predict novel miRNA via MIREAP software (https://sourceforge.net/projects/mireap/). In total, 295 novel miRNAs were identified in the present study (120 novel miRNAs in A1, 79 novel miRNAs in A2, 58 novel miRNAs in A3, 176 novel miRNAs in B1, 95 novel miRNAs in B2, and 70 novel miRNAs in B3) (Table S3). Twenty-two novel miRNAs were coexpressed in the six libraries, accounting for 7.45% of all detected novel miRNAs. This percentage was far below the percentage of coexpressed known miRNAs out of all detected known miRNAs in the six libraries (53.92%), which suggests that the novel miRNAs expressed in each library are more specific. Compared to known miRNAs, most novel miRNAs exhibited low expression levels in all libraries, and this result is consistent with other studies on miRNA sequencing (Yan *et al.* 2015; Zhang *et al.* 2016).

To prove the reliability of novel miRNAs predicted in this study, four novel miRNAs (novel_mir_31, novel_mir_90, novel_mir_98, and novel_mir_9) were randomly selected and confirmed using stem-loop RT-PCR. These four novel miRNAs were all detected in Gui 99 and *fsv1* ovules, suggesting that the results of novel miRNAs are reliable (Figure S1B). The secondary structures of these four novel miRNAs predicted by MIREAP software are also shown in Figure S1C. The primers used for stem-loop RT-PCR are listed in Table S1.

### Differential expression of known miRNA among the three ovule developmental stages in fsv1 and Gui 99 ovules

**Figure 3:**
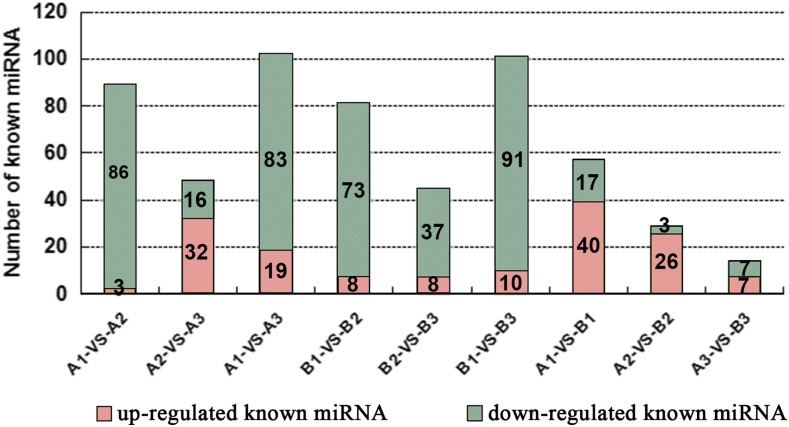
The distribution of upregulated and downregulated known miRNAs that were significantly differentially expressed in each comparison.

In order to identify the known miRNAs with expression levels that changed dynamically among the three ovule developmental stages in *fsv1* and Gui 99 ovules, the TPM was used to normalize the read count of each identified miRNA in each sample. As a result, 89, 48, 102, 81, 45, 101, 57, 29, and 14 known miRNAs were found to be significantly differentially expressed (|fold change log_2_| > 1 and *P*-value <0.05) in the comparisons of A1 *vs.* A2, A2 *vs.* A3, A1 *vs.* A3, B1 *vs.* B2, B2 *vs.* B3, B1 *vs.* B3, A1 *vs.* B1, A2 *vs.* B2, and A3 *vs.* B3, respectively (Figure 3 and Table S4). From stage 1 to stage 2, there were more downregulated known miRNAs than upregulated known miRNAs in Gui 99 and *fsv1* ovules, indicating that the downregulated miRNAs comprised a greater proportion of differentially expressed miRNAs. From stage 2 to stage 3, the upregulated known miRNAs comprised a greater proportion of differentially expressed known miRNAs in Gui 99, whereas the downregulated known miRNAs comprised a greater proportion of differentially expressed miRNAs in *fsv1*.

To elucidate the miRNA regulatory role in rice female fertility, we compared the expression levels of known miRNAs in Gui 99 and *fsv1* ovules at three ovule developmental stages. Fifty-seven known miRNAs were significantly differentially expressed at stage 1 between Gui 99 and *fsv1* (Figure 3 and Table 2). Compared to Gui 99, 40 known miRNAs were upregulated, while 17 known miRNAs were downregulated in *fsv1*. At stage 2, 29 known miRNAs were significantly differentially expressed between Gui 99 and *fsv1*, with 26 known miRNAs upregulated and three known miRNAs downregulated. At stage 3, 14 known miRNAs exhibited significant differential expression between Gui 99 and *fsv1*, with seven known miRNAs upregulated and seven known miRNAs downregulated. During ovule development, the number of differentially expressed known miRNAs between Gui 99 and *fsv1* ovules appears to show a downward trend, which means that the miRNA may play a regulatory role in female fertility mainly at the early ovule developmental stage. At stage 1 and 2, there were more upregulated known miRNAs than downregulated known miRNAs. At stage 3, the same number was shared between upregulated known miRNAs and downregulated known miRNAs.

**Table 2:** Summary of significantly differentially expressed miRNAs between ovules of two rice lines at three developmental stages

miRNA Name	Fold Change	*P*-Value	Significance	Comparison
osa-miR5488	7.82094479	2.78E−06	**	A1 *vs.* B1
osa-miR5836	7.27351589	1.59E−04	**	A1 *vs.* B1
osa-miR5797	3.75281093	6.77E−04	**	A1 *vs.* B1
osa-miR1883a	3.11534253	1.39E−02	*	A1 *vs.* B1
osa-miR5506	2.92473994	8.10E−13	**	A1 *vs.* B1
osa-miR5521	2.75218414	1.76E−05	**	A1 *vs.* B1
osa-miR3980a-3p	2.60798643	6.77E−04	**	A1 *vs.* B1
osa-miR3980b-3p	2.60798643	6.77E−04	**	A1 *vs.* B1
osa-miR166k-5p	2.45950738	1.19E−06	**	A1 *vs.* B1
osa-miR814b	2.26674866	2.68E−02	*	A1 *vs.* B1
osa-miR5491	2.1579006	4.66E−13	**	A1 *vs.* B1
osa-miR5538	2.11475903	3.48E−03	**	A1 *vs.* B1
osa-miR5486	2.07420537	4.73E−92	**	A1 *vs.* B1
osa-miR5499	1.95690004	1.32E−13	**	A1 *vs.* B1
osa-miR5528	1.87094304	5.46E−03	**	A1 *vs.* B1
osa-miR5800	1.85415458	7.35E−16	**	A1 *vs.* B1
osa-miR5792	1.84247953	7.11E−23	**	A1 *vs.* B1
osa-miR5791	1.82323489	7.14E−13	**	A1 *vs.* B1
osa-miR5793	1.72252808	5.36E−58	**	A1 *vs.* B1
osa-miR5818	1.69493794	2.39E−04	**	A1 *vs.* B1
osa-miR5519	1.64534893	1.05E−10	**	A1 *vs.* B1
osa-miR5516a	1.61474232	5.61E−04	**	A1 *vs.* B1
osa-miR5516b	1.61474232	5.61E−04	**	A1 *vs.* B1
osa-miR166h-5p	1.51609091	9.67E−09	**	A1 *vs.* B1
osa-miR1875	1.50973486	2.43E−06	**	A1 *vs.* B1
osa-miR5497	1.36882563	4.14E−177	**	A1 *vs.* B1
osa-miR5487	1.31703111	3.94E−70	**	A1 *vs.* B1
osa-miR5504	1.26689451	3.13E−02	*	A1 *vs.* B1
osa-miR812g	1.21798227	1.13E−02	*	A1 *vs.* B1
osa-miR5806	1.20149479	8.86E−52	**	A1 *vs.* B1
osa-miR812j	1.17941908	3.57E−03	**	A1 *vs.* B1
osa-miR5834	1.11485627	2.29E−02	*	A1 *vs.* B1
osa-miR164a	1.10786112	2.16E−10	**	A1 *vs.* B1
osa-miR164b	1.10786112	2.16E−10	**	A1 *vs.* B1
osa-miR164f	1.10786112	2.16E−10	**	A1 *vs.* B1
osa-miR5490	1.09691878	5.97E−03	**	A1 *vs.* B1
osa-miR162a	1.08242716	1.38E−03	**	A1 *vs.* B1
osa-miR812i	1.05456378	7.00E−03	**	A1 *vs.* B1
osa-miR172a	1.02088245	2.71E−04	**	A1 *vs.* B1
osa-miR172d-3p	1.02088245	2.71E−04	**	A1 *vs.* B1
osa-miR1846e	−1.13753918	9.35E−05	**	A1 *vs.* B1
osa-miR171c-5p	−1.18059947	1.92E−07	**	A1 *vs.* B1
osa-miR444d.3	−1.22500931	4.13E−02	*	A1 *vs.* B1
osa-miR156l-5p	−1.27750872	8.09E−03	**	A1 *vs.* B1
osa-miR2093-3p	−1.37698782	4.25E−02	*	A1 *vs.* B1
osa-miR5179	−1.48680553	9.72E−10	**	A1 *vs.* B1
osa-miR6250	−1.63998183	2.12E−02	*	A1 *vs.* B1
osa-miR167a-3p	−1.70370182	1.08E−37	**	A1 *vs.* B1
osa-miR1846a-5p	−1.74316161	6.99E−04	**	A1 *vs.* B1
osa-miR1846b-5p	−1.74316161	6.99E−04	**	A1 *vs.* B1
osa-miR1849	−1.79214958	4.86E−02	*	A1 *vs.* B1
osa-miR1852	−1.92971675	2.93E−02	*	A1 *vs.* B1
osa-miR1847.1	−2.22516085	3.28E−02	*	A1 *vs.* B1
osa-miR1850.2	−2.37711208	1.88E−02	*	A1 *vs.* B1
osa-miR6253	−2.55769805	1.11E−03	**	A1 *vs.* B1
osa-miR1432-5p	−2.59423894	2.83E−15	**	A1 *vs.* B1
osa-miR5074	−3.22516085	9.82E−03	**	A1 *vs.* B1
osa-miR394	10.27233818	3.59E−27	**	A2 *vs.* B2
osa-miR5505	7.25738784	5.66E−04	**	A2 *vs.* B2
osa-miR159a.2	4.3917989	1.79E−16	**	A2 *vs.* B2
osa-miR528-5p	3.98724708	0	**	A2 *vs.* B2
osa-miR160d-5p	3.9767614	6.51E−12	**	A2 *vs.* B2
osa-miR160a-5p	3.89429924	3.66E−11	**	A2 *vs.* B2
osa-miR160b-5p	3.89429924	3.66E−11	**	A2 *vs.* B2
osa-miR160c-5p	3.89429924	3.66E−11	**	A2 *vs.* B2
osa-miR408-3p	3.89396937	2.30E−04	**	A2 *vs.* B2
osa-miR397b	3.26593815	7.01E−03	**	A2 *vs.* B2
osa-miR160e-5p	3.14918147	1.71E−24	**	A2 *vs.* B2
osa-miR5800	3.02922882	1.38E−05	**	A2 *vs.* B2
osa-miR166k-5p	2.97643153	2.11E−02	*	A2 *vs.* B2
osa-miR444f	2.80700136	1.94E−03	**	A2 *vs.* B2
osa-miR160d-3p	2.80650653	3.64E−02	*	A2 *vs.* B2
osa-miR1425-5p	2.51414398	2.54E−28	**	A2 *vs.* B2
osa-miR408-5p	2.45061011	4.84E−17	**	A2 *vs.* B2
osa-miR2873b	1.92231362	1.27E−03	**	A2 *vs.* B2
osa-miR160a-3p	1.71364452	2.62E−02	*	A2 *vs.* B2
osa-miR160b-3p	1.71364452	2.62E−02	*	A2 *vs.* B2
osa-miR5519	1.68119555	8.62E−04	**	A2 *vs.* B2
osa-miR531a	1.61413634	6.33E−06	**	A2 *vs.* B2
osa-miR531b	1.61413634	6.33E−06	**	A2 *vs.* B2
osa-miR531c	1.61413634	6.33E−06	**	A2 *vs.* B2
osa-miR1319b	1.57223919	2.52E−02	*	A2 *vs.* B2
osa-miR5486	1.35594481	1.21E−12	**	A2 *vs.* B2
osa-miR1868	−1.01635756	3.96E−02	*	A2 *vs.* B2
osa-miR5817	−1.93016219	3.15E−02	*	A2 *vs.* B2
osa-miR2863c	−2.51512469	1.17E−02	*	A2 *vs.* B2
osa-miR2865	6.67398035	7.41E−03	**	A3 *vs.* B3
osa-miR6253	2.24142558	6.94E−03	**	A3 *vs.* B3
osa-miR5490	2.18911734	3.65E−02	*	A3 *vs.* B3
osa-miR394	2.13449408	2.81E−04	**	A3 *vs.* B3
osa-miR812p	1.30852693	1.82E−02	*	A3 *vs.* B3
osa-miR1846e	1.13457759	2.13E−02	*	A3 *vs.* B3
osa-miR5497	1.11526721	1.15E−12	**	A3 *vs.* B3
osa-miR171c-5p	−1.102933	7.47E−03	**	A3 *vs.* B3
osa-miR5150-3p	−1.9809604	7.86E−03	**	A3 *vs.* B3
osa-miR5485	−2.56592291	1.19E−04	**	A3 *vs.* B3
osa-miR396a-5p	−3.15067993	1.24E−02	*	A3 *vs.* B3
osa-miR396b-5p	−3.15067993	1.24E−02	*	A3 *vs.* B3
osa-miR5815	−3.15067993	1.24E−02	*	A3 *vs.* B3
osa-miR812f	−6.65492239	8.23E−03	**	A3 *vs.* B3

In this study, the significantly differentially expressed miRNAs between two samples were labeled with ** or *. If the |fold change log_2_| > 1 and *P*-value <0.01, miRNAs were labeled with **. If |fold change log_2_| > 1 and *P*-value between <0.01 and <0.05, miRNAs were labeled with *.

### Determination of coherent target genes and potential function analysis of known miRNAs

To figure out the potential function of miRNAs, the identification of their target genes is particularly important. Using psRobot and TargetFinder software, we predicted the potential target genes of all differentially expressed known miRNAs in all comparisons. Comparing the expression profiles of detected known miRNAs with the transcriptome profiles of their corresponding predicted targets, which we obtained from the same experimental system in Yang *et al.* (2016), we found that some miRNAs exhibited the same expression trends as their corresponding predicted targets in the same comparison, while some miRNAs exhibited opposite expression trends as their corresponding predicted targets in the same comparison. As miRNAs usually negatively regulate the expression of their target genes, the predicted targets that exhibited the opposite expression trends as the miRNAs in the same comparison were defined as coherent target genes. The coherent target genes were the focus of this study (Table S5). In accordance with a previous report (Shin *et al.* 2009), several genes with similar function are generally targeted by the same miRNA. For instance, *ARF10* and *ARF22* (of the ARF family) were targeted by miR172 in the comparison of A1 *vs.* A2, and both of them could respond to the auxin level in plants. Meanwhile, miRNAs that target the same gene usually belong to the same family. For instance, in the comparison of A1 *vs.* B1, *OMTN4* was cotargeted by miR164a, miR164b, and miR164f. In the comparison of A2 *vs.* B2, *ARF13* was cotargeted by miR160a-5p, miR160b-5p, miR160c-5p, miR160d-5p, and miR160e-5p.

**Figure 4:**
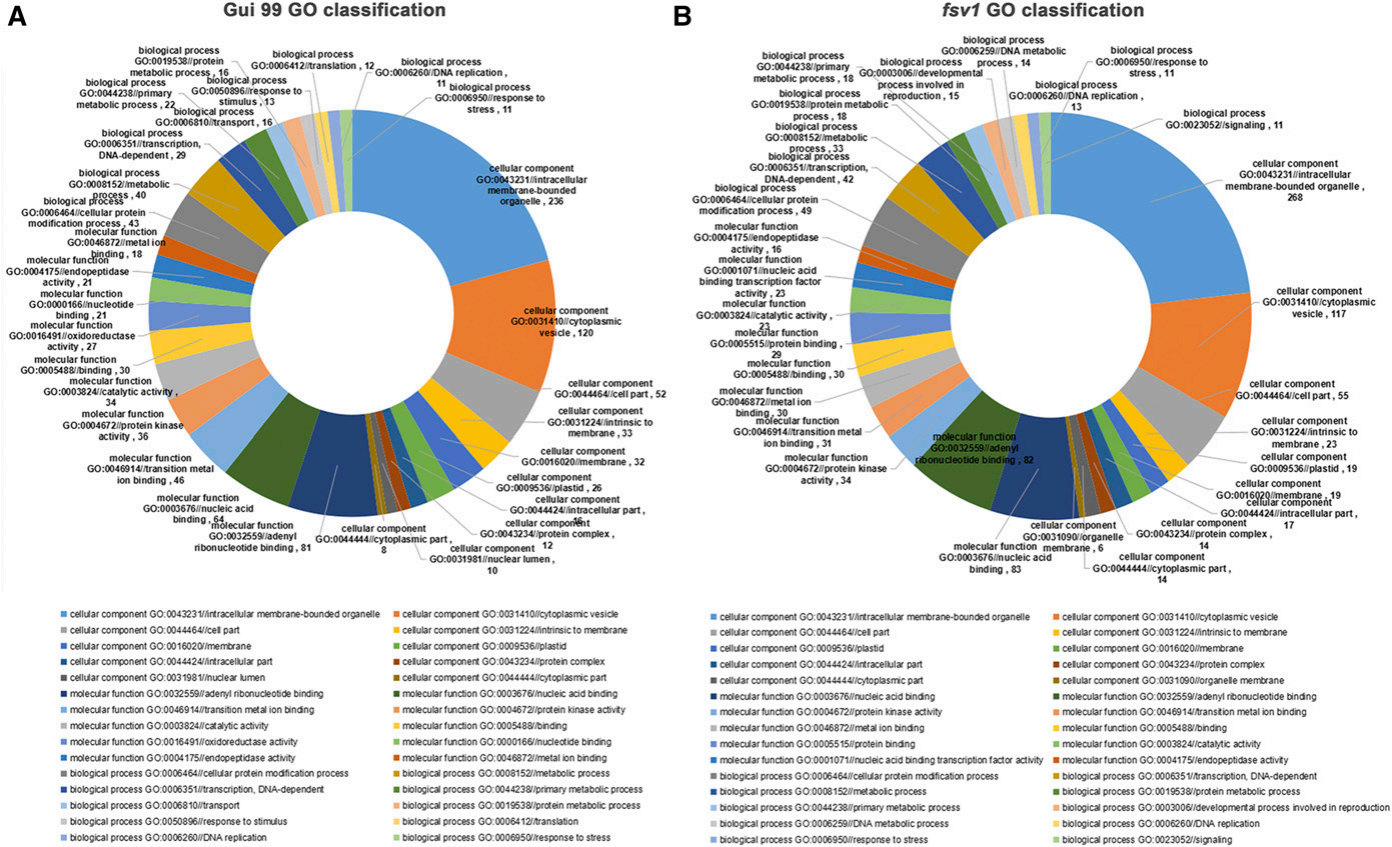
(A) GO classifications of coherent target genes of known miRNAs that were differentially expressed in different comparisons of Gui 99. (B) GO classifications of coherent target genes of known miRNAs that were differentially expressed in different comparisons of *fsv1*. The top 10 most abundant GO terms in each category are listed.

To further investigate the regulatory functions of miRNAs, all predicted coherent target genes were annotated to different functional GO terms using Blast2GO (version 2.3.5) (http://www.blast2go.org/). We classified the functional information of the coherent target genes of differentially expressed known miRNAs among the three ovule developmental stages in *fsv1* and Gui 99 ovules using the WEGO website (http://wego.genomics.org.cn/). As we can see from Table S5, the coherent target genes of one miRNA could be annotated to diverse functional GO terms, while one functional GO term could be annotated by coherent target genes of different miRNAs. In Gui 99, coherent target genes of differentially expressed known miRNAs in comparisons of A1 *vs.* A2, A2 *vs.* A3, and A1 *vs.* A3 were classified into 336 GO terms. The most abundant GO terms in the cellular component, molecular function, and biological process categories were intracellular membrane-bounded organelle (GO:0043231), adenyl ribonucleotide binding (GO:0032559), and cellular protein modification process (GO:0006464), respectively (Figure 4A). In *fsv1*, coherent target genes of differentially expressed known miRNAs in comparisons of B1 *vs.* B2, B2 *vs.* B3, and B1 *vs.* B3 were classified into 190, 128, and 226 GO terms, respectively. The most abundant GO term in the molecular function category was nucleic acid binding (GO:0003676), which was different from Gui 99. The most abundant GO terms in the cellular component, molecular function, and biological process categories were intracellular membrane-bounded organelle (GO:0043231), nucleic acid binding (GO:0003676), and cellular protein modification process (GO:0006464), respectively (Figure 4B and Table S5).

**Figure 5:**
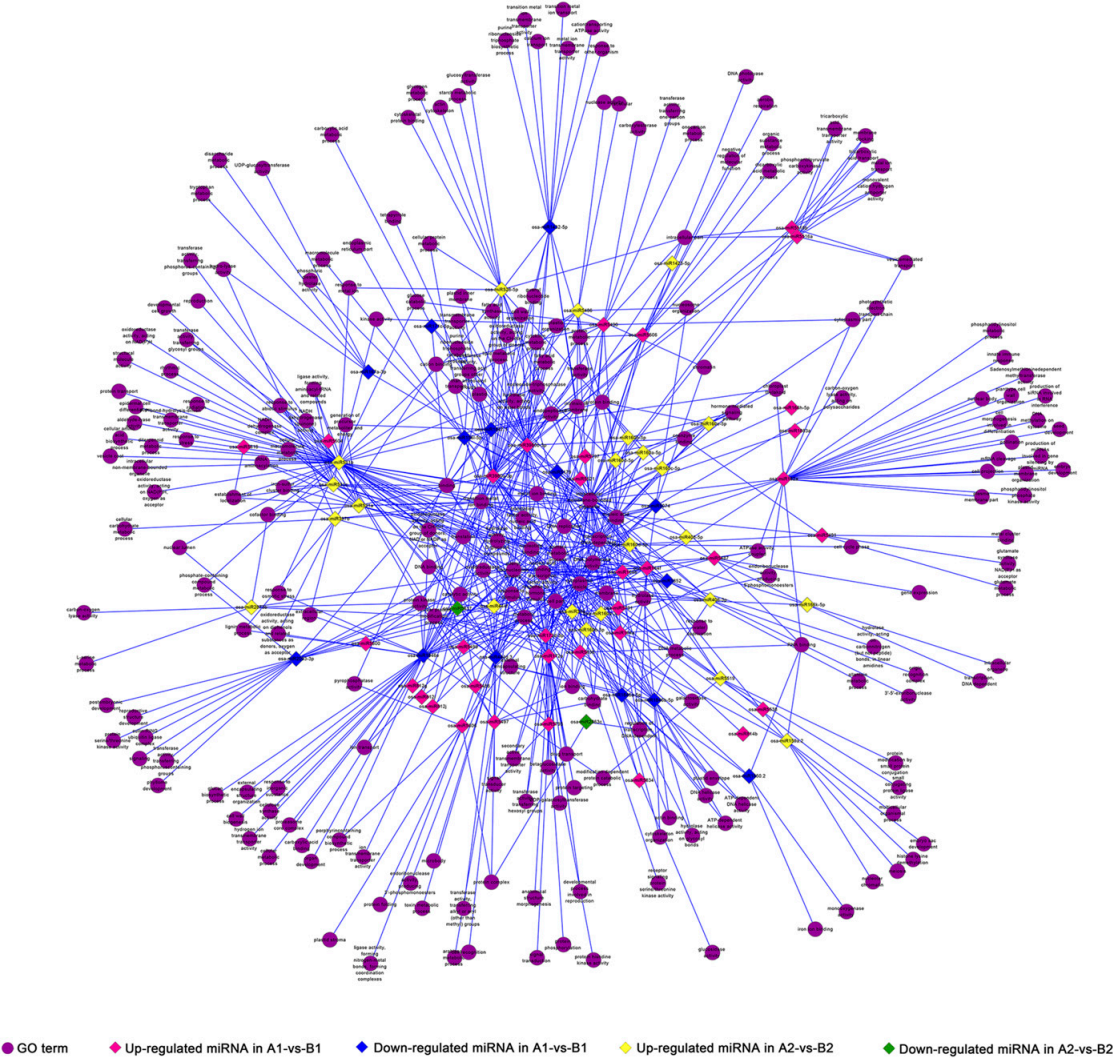
miRNA GO term network.

In order to figure out the regulating effects of known miRNAs on female gametophyte fertility, we focused on the functions of coherent target genes of known miRNAs that were differentially expressed in comparisons of A1 *vs.* B1 and A2 *vs.* B2 (Figure 5). As Table S5 shows, the coherent target genes of known miRNAs that were differentially expressed in these two comparisons were mainly annotated to several GO terms, such as cell part (GO:0044464), intracellular membrane-bounded organelle (GO:0043231), cytoplasmic vesicle (GO:0031410), and nucleic acid binding (GO:0003676). On the other hand, some targets were classified into specific GO terms. For instance, only coherent target genes of known miRNAs that were differentially expressed in comparison A1 *vs.* B1 were annotated to reproduction (GO:0003006), signal transduction (GO:0007165), calcium ion transport (GO:0006816), and ATP-dependent DNA helicase activity (GO:0004003). Only coherent target genes of known miRNAs that were differentially expressed in comparison A2 *vs.* B2 were annotated to embryo sac development (GO:0009553) and protein transport (GO:0015031).

At stage 1, 251 coherent target genes of known miRNAs that were differentially expressed in comparison A1 *vs.* B1 were annotated to 189 GO terms. According to the results of the GO enrichment analysis, the enriched GO terms were cellular macromolecule biosynthetic process (GO:0034645), macromolecule biosynthetic process (GO:0009059), DNA replication (GO:0006260), endopeptidase activity (GO:0004175), and DNA polymerase activity (GO:0034061) (Table S6). Some GO terms were only annotated by coherent target genes of upregulated known miRNAs in comparison A1 *vs.* B1, such as vesicle-mediated transport (GO:0016192), kinase activity (GO:0016301), and protein phosphorylation (GO:0006468) (Table S5).

At stage 2 (A2 *vs.* B2), there were 161 coherent target genes of known miRNAs that were differentially expressed in comparison A2 *vs.* B2. A total of 159 were targeted by upregulated miRNAs and two of them were targeted by downregulated miRNAs, and they were annotated to 111 and six GO terms, respectively. According to the results of the GO enrichment analysis, the enriched GO terms were lignin metabolic process (GO:0009808); phenylpropanoid metabolic process (GO:0009698); secondary metabolic process (GO:0019748); oxidoreductase activity, acting on diphenols and related substances as donors, oxygen as acceptor (GO:0016682); oxidoreductase activity, acting on diphenols and related substances as donors (GO:0016679); and oxidoreductase activity (GO:0016491) (Table S6). Compared with the Gui 99 ovule, most of the differentially expressed miRNAs were upregulated in the *fsv1* ovule. The targets of these upregulated miRNAs were annotated to GO terms such as signal transducer activity (GO:0004871), response to hormone stimulus (GO:0009725), intracellular membrane-bounded organelle (GO:0043231), and cytoplasmic vesicle (GO:0031410), which indicated that the functions of these targets were diversified. As miRNAs can inhibit the expression their target genes, these targets may be related to female gametophyte formation and be inhibited by the upregulated miRNAs in *fsv1* relative to the Gui 99 ovule (Table S5).

At stage 3, the coherent target genes of known miRNAs that were differentially expressed in comparison A3 *vs.* B3 were annotated to 62 GO terms. Based on the results of GO enrichment analysis, there were 33 enriched GO terms, such as DNA replication (GO:0006260), DNA polymerase activity (GO:0034061), and transferase activity (GO:0016740) (Table S6).

A previous study has shown that the target genes of miRNAs that were differentially expressed between the rice male sterile line and its maintainer line were mainly annotated to binding (GO:0005488) and catalytic activity (GO:0003824) in the molecular function category and metabolic process (GO:0008152) in the biological process category (Yan *et al.* 2015). In our study, many coherent target genes of differentially expressed known miRNAs between *fsv1* and Gui 99 were also annotated to these GO terms. In addition, of all the differentially expressed known miRNAs between *fsv1* and Gui 99, nine known miRNAs (miR1432-5p, miR160a-3p, miR160b-3p, miR167a-3p, miR3980a-3p, miR3980b-3p, miR397b, miR528-5p, and miR5793) exhibited significant differential expression between the rice male sterile line and its maintainer line. The coherent target genes of these known miRNAs were annotated to 17 GO terms. Cytoplasmic vesicle (GO:0031410) and transition metal ion binding (GO:0046914) were the most abundant GO terms in the cellular component and molecular function categories. The results of GO annotation indicated that genes which were targeted by these nine known miRNAs had different functions and they were involved in many different biological processes. The effects of these nine known miRNAs on regulating male and female fertility in rice remain to be elucidated.

### Many predicted miRNA targets were the transcription factor genes

Transcription factors are important regulators of gene expression. In previous studies, many miRNA-targeted genes in plants that have been found to be the transcription factor genes also play an important role in developmental processes (Rubio-Somoza and Weigel 2011; Xie *et al.* 2015; Shu *et al.* 2016). In our study, nearly 10% coherent target genes of differentially expressed known miRNAs in all comparisons were found to belong to transcription factor families (Table S7). In Gui 99, 75 transcription factor genes were targeted by 57 differentially expressed known miRNAs in the comparisons of A1 *vs.* A2, A2 *vs.* A3, and A1 *vs.* A3, and many of them were ARF, NAC, and GRF family members. In *fsv1*, 65 transcription factor genes were targeted by 50 differentially expressed known miRNAs of comparisons of B1 *vs.* B2, B2 *vs.* B3, and B1 *vs.* B3, and many of them were targeted by miR160, miR164, miR171, and miR172. The members of the C3H, GeBP, M-type, TALE, and ZF-HD families were only targeted by differentially expressed known miRNAs in the Gui 99 ovule, while the members of CO-like, HD-ZIP, and HSF families were only targeted by differentially expressed known miRNAs in the *fsv1* ovule.

**Figure 6:**
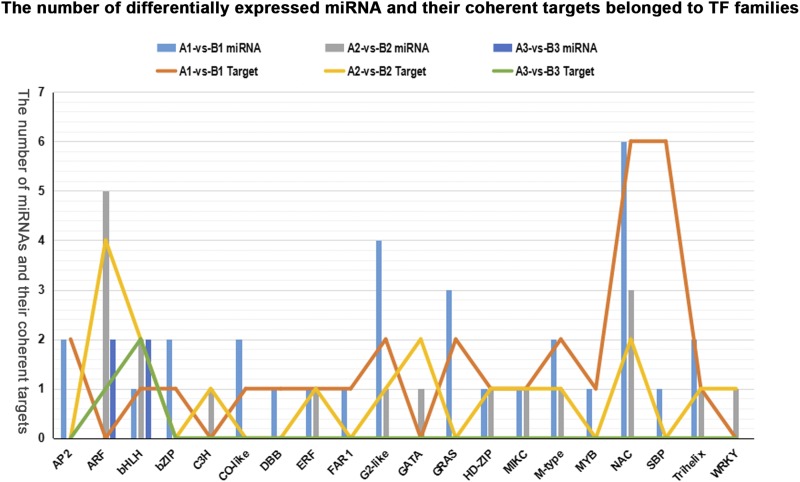
The number of known miRNAs that were differentially expressed in comparisons A1 *vs.* B1, A2 *vs.* B2, and A3 *vs.* B3 and their coherent target genes belonging to transcription factor families.

To explore the regulatory role of miRNA in rice female gametophyte abortion, we paid more attention to transcription factor family members that were targeted by the significantly differentially expressed miRNAs in the pairs A1 *vs.* B1 and A2 *vs.* B2 (Figure 6). In the comparison of A1 *vs.* B1, 30 coherent target genes of 18 significantly differentially expressed known miRNAs were found to belong to 16 transcription factor families (such as AP2, NAC, and SBP). The expression levels of 12 known miRNAs (miR5793, miR5521, miR5519, miR5491, miR3980a-3p, miR3980b-3p, miR172a, miR172d-3p, miR166k-5, miR164a, miR164b, and miR164f) were significantly upregulated in *fsv1* ovules, while those of six known miRNAs (miR5074, osa-miR444d.3, miR1850.2, miR156l-5p, miR171c-5p, and miR1846e) were downregulated in *fsv1* ovules. These miRNAs may be involved in the process of MMC meiosis by regulating expressions of transcription factor genes in rice. In the comparison of A2 *vs.* B2, 18 coherent target genes of 12 significantly differentially expressed known miRNAs (miR160a-3p, miR160b-3p, miR160a-5p, miR160b-5p, miR160c-5p, miR160d-5p, miR160e-5p, miR166k-5p, miR394, miR444f, miR528-5p, and miR531b) were found to belong to the families of ARF, bHLH, C3H, ERF, G2-like, GATA, HD-ZIP, MIKC, M-type, NAC, Trihelix, and WRKY, and these 12 known miRNAs were all significantly upregulated in *fsv1* ovules. The upregulation of these 12 known miRNAs may function in regulating the fertile female gametophyte formation by silencing the expression of transcription factor genes related to functional megaspore mitosis.

In the A3 *vs.* B3 comparison, four known miRNAs were significantly differentially expressed between *fsv1* and Gui 99 ovules, while three coherent target genes of these four known miRNAs were found to belong to the ARF and bHLH families (Figure 6). These known miRNAs might be important regulators contributing to the structure differentiation of Gui 99 and *fsv1* ovules.

### Validation of sequencing results by quantitative real-time PCR analysis

Six known miRNAs with different expression profiles were selected randomly, and their expression profiles were used to verify the sequencing results by quantitative real-time PCR analysis. As a result, the known miRNA expression patterns determined by quantitative real-time PCR were basically consistent with the sequencing results, confirming the accuracy of sequencing results. The results of the quantitative real-time PCR are shown in Figure S1A and the primer sequences are available in Table S1.

## Discussion

In recent studies, small RNA pathways were reported to play important regulatory roles in the developmental process of plants (Chen 2009; Fei *et al.* 2016). The Argonaute protein and miRNA are key components of small RNA pathways. Many Argonaute proteins, such as AGO9 and AGO104, have been proved to be essential for the process of gamete formation in *Arabidopsis* and maize (Olmedo-Monfil *et al.* 2010; Singh *et al.* 2011). miRNAs, as another key component of small RNA pathways, have been certified to participate in the reproductive process of plants. The relationship between miRNAs and male gametophyte fertility have been extensively studied in several plant species (Wei *et al.* 2013; Yang *et al.* 2013; Zhang *et al.* 2016). However, few studies have reported on the relationship between the miRNAs and female gametophyte fertility in rice (Wu *et al.* 2006). In this study, we present the application of high-throughput sequencing technology to identify miRNAs expressed in the rice ovule during ovule development, and report a comprehensive miRNA transcriptome analysis of rice high frequency female-sterile line and wild-type line ovules. In total, 522 known miRNAs were identified in the developing ovule in rice, more than the number of miRNAs detected in the cotton ovule (Xie *et al.* 2015). By comparing all miRNAs detected in this study with miRNAs detected in rice pollen and sporophytic tissues [root and leaf; pollen, root, and leaf data from Additional File 1 of Wei *et al.* (2011)], ∼70% miRNAs were exclusively expressed in the rice ovule, suggesting that these exclusively expressed miRNAs may play specific regulatory roles in female reproductive tissue (Figure S2 and Table S8). Moreover, in a differential expression analysis of our study, 100 known miRNAs exhibited significant differential expression between *fsv1* and Gui 99. During ovule development, numerous miRNAs exhibited distinct patterns of expression in Gui 99 and *fsv1* ovules. These results suggest that miRNA may be a key regulator involved in rice female gametophyte abortion. Additionally, compared with Gui 99, the number of upregulated miRNAs was significantly larger than downregulated miRNAs in *fsv1*, suggesting that miRNA may control female fertility primarily through reducing the expression of genes related to the formation of the fertile female gametophyte.

The development of the ovule is a complex process involving diverse biological pathways (Kubo *et al.* 2013). Previous studies have demonstrated that the expression variations of some genes related to key biological pathways in the ovule could also affect female gametophyte fertility (Olmedo-Monfil *et al.* 2010; Tucker *et al.* 2012). miRNAs, as one kind of the most important regulators of gene expression, have been proved to be involved in numerous biological pathways in the ovule (Xie *et al.* 2015). In *Arabidopsis*, a group of ATP-dependent helicase genes have been found to be specifically enriched in MMCs. Mutation of the RNA helicase gene, *MEM*, results in the defects of megasporogenesis and megagametogenesis, suggesting that RNA helicase genes play an essential role in the formation of the female gametophyte (Schmidt *et al.* 2011). In this study, miR164a, miR164b, miR164f, and miR531b were significantly upregulated in the *fsv1* ovule at stage 1 and stage 2. All of them were identified to target ATP-dependent RNA helicase genes. The upregulation of these miRNAs may be associated with female gametophyte abortion. Moreover, miR444d.3, miR2093-3p, and miR531b, which were significantly differentially expressed in the ovules of *fsv1* and Gui 99, were identified to target important genes related to the calcium/calmodulin signaling pathway. In lettuce, it has been demonstrated that the distribution and concentration of calcium, which both play an important role in megasporogenesis, are closely associated with megaspore degeneration (Qiu *et al.* 2008). These differentially expressed miRNAs may affect the fertility of female gametophyte via affecting the calcium/calmodulin signaling pathway.

As one of the most important phytohormones, auxin is involved in many plant biological processes, such as root development, leaf pattern formation, and flower development (Teale *et al.* 2006). In past years, many researchers have revealed the significant roles of auxin in the development of the female gametophyte. The distribution and specification of member cells (including the egg cell, synergid cells, central cell, and antipodal cells) in the female gametophyte could be determined by auxin concentration (Pagnussat *et al.* 2009). The expression changes of some auxin-related genes in ovules could affect the formation of fertile female gametophyte (Panoli *et al.* 2015). In rice, 5% of miRNAs have been proved to be sensitive to auxin. These miRNAs are likely to be early hormone response factors during a developmental event (Meng *et al.* 2009). Thus, miRNAs could participate in female gametophyte formation by regulating auxin-related genes. For example, ARFs are DNA-binding proteins that are specifically expressed in plants. They could combine with the promotor region of auxin-responsive genes *Aux*/*IAA*, *SAUR*, and *GH3*, and activate or repress the expression of early auxin-response genes (Abel and Theologis 1996; Ulmasov *et al.* 1999; Hagen and Guilfoyle 2002). In *Arabidopsis*, miR160 could regulate the expressions of many ARF family genes (such as *ARF10*, *ARF16*, and *ARF17*). The expression variation of miR160 could lead to the abnormal expression of *ARF17*, *YDK1*/*GH3.2*, *GH3.3*, *GH3.5*, and *DFL1*/*GH3.6*, and result in diverse reproductive developmental defects, such as petal size shrinking, flowering time promoting, fertility reducing, and so on (Mallory *et al.* 2005). Moreover, in previous research, miR167 was proved to target the *ARF6* and *ARF8* transcripts in *Arabidopsis*, and it functions in the regulation of female and male reproduction by regulating the expression of *ARF6* and *ARF8* (Wu *et al.* 2006), further suggesting that miRNAs could affect female and male gametophyte development by regulating the auxin-related genes. In this study, the targets of miR160a-5p, miR160b-5p, miR160c-5p, miR160d-5p, miR160e-5p, and miR408-3p were predicted to be genes (such as *ARF10*, *ARF13*, *BG1*, and *PIN1c*) related to auxin response and transport, and these miRNAs were significantly differentially expressed between the ovules of *fsv1* and Gui 99. These miRNAs may function in regulating female gametophyte fertility by regulating the auxin signaling pathway in rice.

Transcription factors are important transcriptional regulatory elements. Many studies have demonstrated the important function of transcription factors in the female reproductive process (Klucher *et al.* 1996; Baker *et al.* 1997; Wu *et al.* 2006). In the cotton ovule, transcription factors occupy a large portion of identified miRNAs targets, indicating miRNAs that actively targeted the transcription factor genes were involved in plant ovule development (Xie *et al.* 2015). Previous studies have also shown that many miRNAs (such as miR156, miR160, miR164, miR166, and miR172) are associated with flower development by regulating expression of the transcription factor genes (Aukerman and Sakai 2003; Achard *et al.* 2004; Wu *et al.* 2006; Oh *et al.* 2008; Shikata *et al.* 2009; Luo *et al.* 2013). For example, SBP-box genes, which are targeted by miR156 or miR157, play a significant role in regulating the differentiation of the flower organ at the reproductive phase (Shikata *et al.* 2009; Yamaguchi *et al.* 2009). In tomato, miR156 mediates the cleavage of SBP-box genes which function as regulator of the normal development of gynoecia (Silva *et al.* 2014). In our results, miR156l-5p, which was predicted to target six members (*OsSPL2*, *OsSPL3*, *OsSPL11*, *OsSPL12*, *OsSPL13*, and *OsSPL19*) of the SBP family, exhibited significant differential expression between ovules of Gui 99 and *fsv1* at stage 1, suggesting a potential role of miR156 in the regulation of fertile female gametophyte formation. In rice, miR172 targets members of the AP2 family. Terribly flawed flower organs and reduced fertility existed in the plants with overexpressed miR172 (Zhu *et al.* 2009). In the present study, the expression of miR172a was significantly increased in *fsv1* ovules at the meiosis stage. The upregulation of miR172 may be one of the factors leading to *fsv1* female abortion. In addition, HD-ZIP III family genes, such as *PHB*, *PHV*, and *CNA*, have been proved to play a significant role in regulating female gametophyte formation. The *phb-13phv-11cna-2* mutant exhibits ovule developmental defects, and results in the reduction of female fertility (Prigge *et al.* 2005). Zhou *et al.* (2015) have found that miR165/166 could regulate the transcripts of the HD-ZIP III family by combining with AGO1 and AGO10. Thus, significantly upregulated miR166k-5p and miR166h-5p in the *fsv1* ovule may be involved in fertile female gametophyte formation by regulating the expression of HD-ZIP III family genes. Furthermore, MADS-box genes are crucial for the differentiation of flower organs (Dreni *et al.* 2007; Li *et al.* 2010, 2011a,b; Yun *et al.* 2013). The abnormal expressions of some MADS-box genes, such as *OsMADS3*, *OsMADS13*, *OsMADS16*, and *OsMADS58*, could lead to a developmental disorder in rice flower organs (Dreni *et al.* 2007; Li *et al.* 2011a; Yun *et al.* 2013). In this study, *OsMADS98*, *SPW1*, and *MADS57*, which belong to the MADS-box family, were targeted by three differentially expressed miRNAs (osa-miR5521, osa-miR5179, and osa-miR444d.3) between the Gui 99 and *fsv1* ovules. These miRNAs may contribute to the formation of fertile female gametophyte.

In summary, we provide comprehensive information about changes in miRNA expression profiles of rice ovule at different developmental stages, and identify many miRNAs that exhibited significant differential expression between a high frequency female-sterile rice line and a wild-type rice line during ovule development. By combining with transcriptome information, the regulatory network of female fertility-related miRNAs and its coherent target genes were revealed. The functional analysis of coherent target genes of these miRNAs revealed that these miRNAs are involved in many biological pathways, such as phytohormone regulation, DNA replication, and transcriptional regulation. These results will help us better understand the regulatory roles of miRNAs in the fertile female gametophyte formation of rice.

## Supplementary Material

Supplemental material is available online at www.g3journal.org/lookup/suppl/doi:10.1534/g3.117.040808/-/DC1.
