# Supplementary figures and images for: Genetic Subtraction Profiling Identifies Candidate miRNAs Involved in Rice Female Gametophyte Abortion
Source: G3 (Bethesda). 2017 May 19;7(7):2281–93. doi: 10.1534/g3.117.040808 (PMC5499135; doi:10.1534/g3.117.040808)

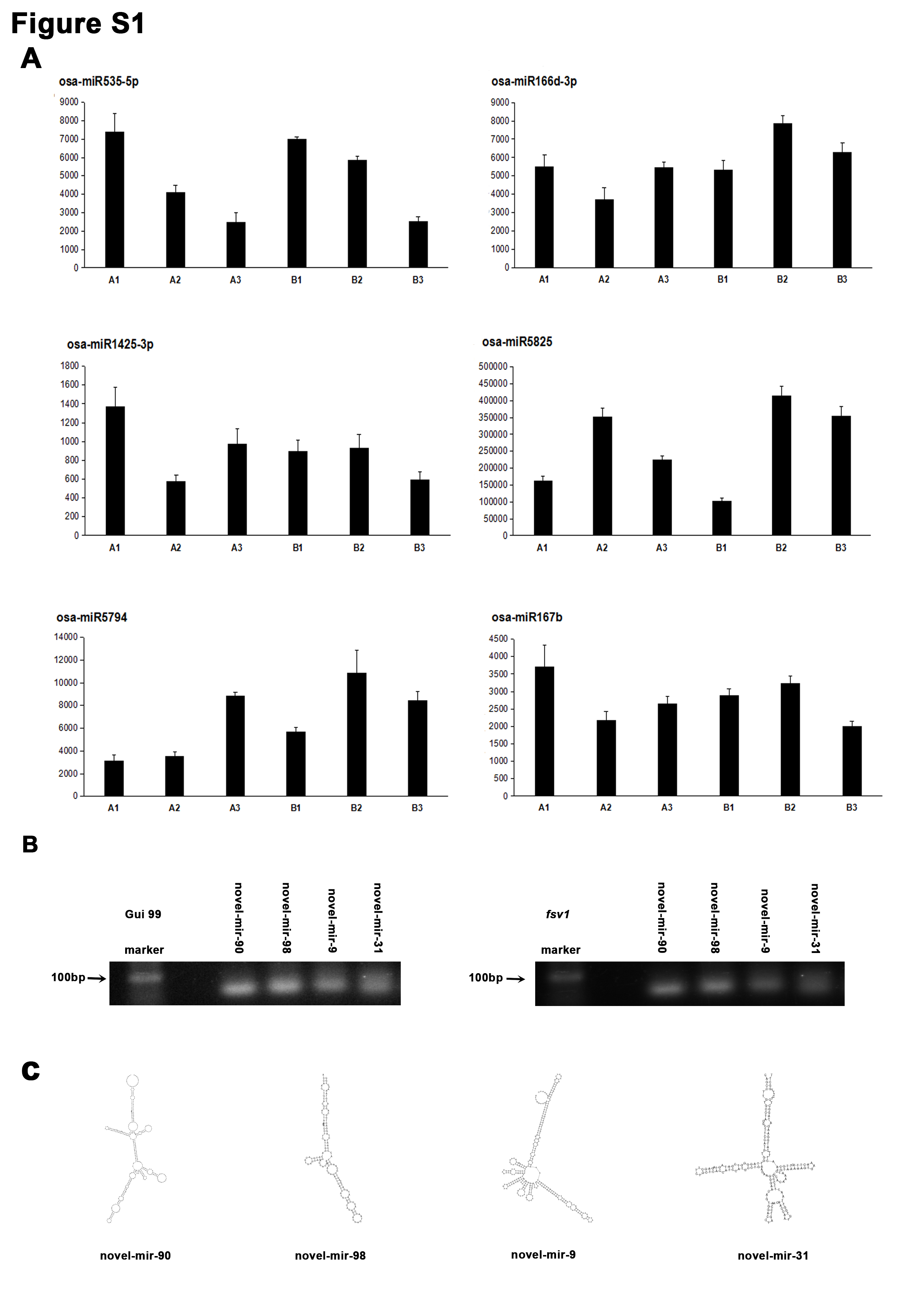

Supplement: Supplementary file 1 [file 2281FigureS1.tif]

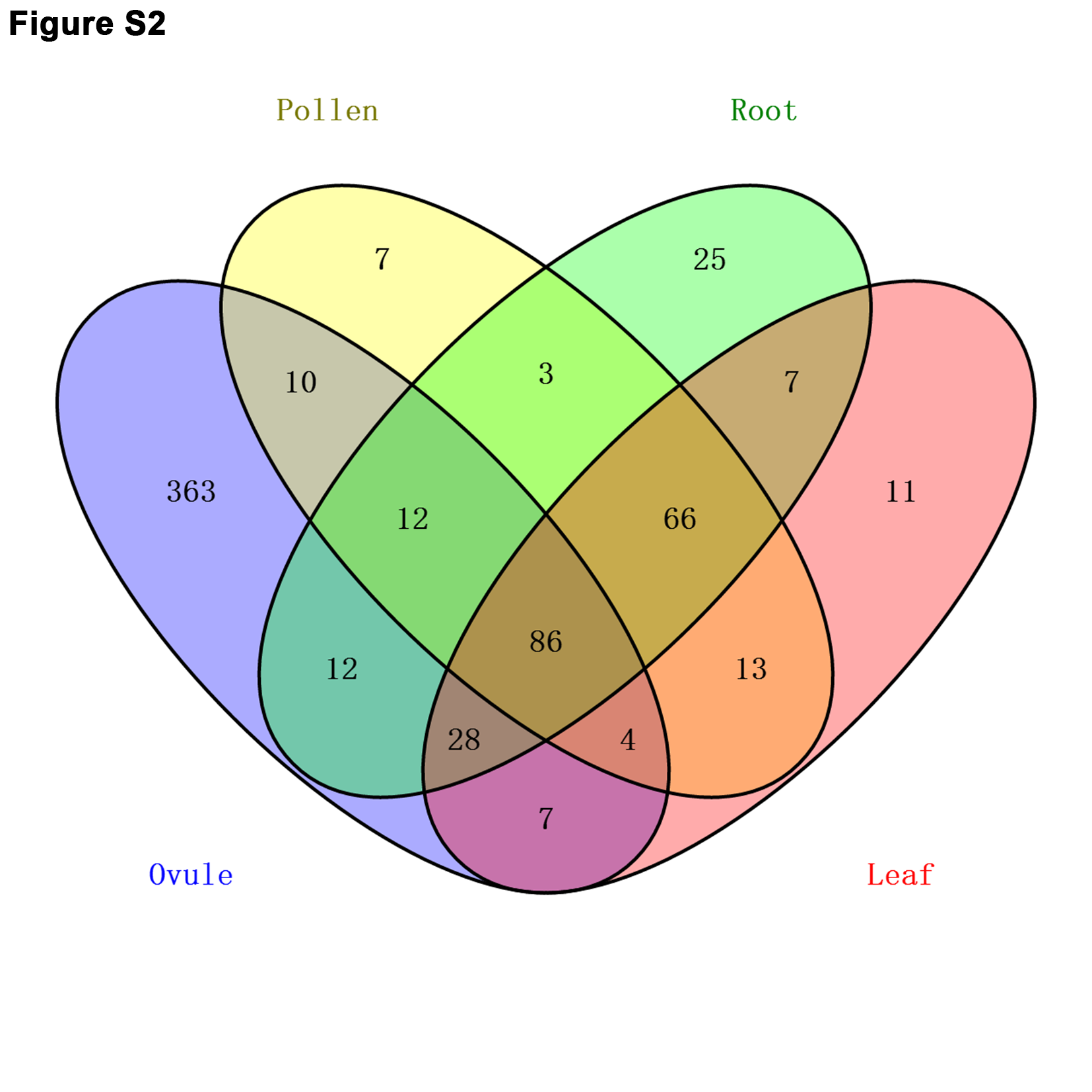

Supplement: Supplementary file 2 [file 2281FigureS2.tif]
